# Treatment of Wilms Tumor in Sub-Saharan Africa: Results of the Second French African Pediatric Oncology Group Study

**DOI:** 10.1200/JGO.18.00204

**Published:** 2019-09-05

**Authors:** Atteby Jean-Jacques Yao, Claude Moreira, Fousseyni Traoré, Sonia Kaboret, Angele Pondy, Mbolanirina Lala Rakotomahefa Narison, Koffi M. Guedenon, Brenda Mallon, Catherine Patte

**Affiliations:** ^1^ Hôpital de Treichville, Abidjan, Ivory Coast; ^2^ Hôpital Aristide Le Dantec, Université Cheikh Anta Diop de Dakar, Dakar, Senegal; ^3^ Hôpital Gabriel Touré, Bamako, Mali; ^4^ Hopital Charles de Gaulle, Centre Hospitalier Universitaire Pédiatrique, Ouagadougou, Burkina Faso; ^5^ Centre Mère et Enfant, Fondation Chantal Biya, Yaoundé, Cameroun; ^6^ Université Joseph Ravoahangy Andrianavalona, Antananarivo, Madagascar; ^7^ Université Sylvanus Olympio, Lomé, Togo; ^8^ Groupe Franco-Africain d'Oncologie Pédiatrique, Gustave Roussy, Villejuif, France

## Abstract

**PURPOSE:**

Multidisciplinary management of Wilms tumor has been defined through multicenter prospective studies and an average expected patient cure rate of 90%. In sub-Saharan Africa, such studies are uncommon. After the encouraging results of the first Groupe Franco-Africain d'Oncologie Pédiatrique (GFAOP) study, we report the results of the GFAOP-NEPHRO-02 study using an adaptation of the International Society of Paediatric Oncology 2001 protocol.

**PATIENTS AND METHODS:**

From April 1, 2005, to March 31, 2011, seven African units participated in a nonrandomized prospective study. All patients who were referred with a clinical and radiologic diagnosis of renal tumor were screened. Those older than age 6 months and younger than 18 years with a unilateral tumor previously untreated were pre-included and received preoperative chemotherapy. Patients with unfavorable histology or with a tumor other than Wilms, or with a nonresponding stage IV tumor were excluded secondarily.

**RESULTS:**

Three hundred thirteen patients were initially screened. Two hundred fifty-seven patients were pre-included and 169 with histologic confirmation of intermediate-risk nephroblastoma were registered in the study and administered postoperative treatment. Thirty-one percent of patients were classified as stage I, 38% stage II, 24% stage III, and 7% stage IV. Radiotherapy was not available for any stage III patients. Three-year overall survival rate was 72% for all study patients and 73% for those with localized disease.

**CONCLUSION:**

It was possible to conduct sub-Saharan African multicenter therapeutic studies within the framework of GFAOP. Survival results were satisfactory. Improvements in procedure, data collection, and outcome are expected in a new study. Radiotherapy is needed to reduce the relapse rate in patients with stage III disease.

## INTRODUCTION

Wilms tumor (WT) accounts for 5.9% of childhood cancers and affects one in every 10,000 children worldwide before the age of 15 years.^1^ Treatment of this rapidly growing tumor has benefited from major therapeutic advances, with an average of 90% of patients achieving cure in the largest series. Numerous multicenter clinical trials conducted by the International Society of Paediatric Oncology (SIOP)^2-8^ in Europe and by the National Wilms Tumor Study Group^9-10^ in the United States have enabled the definition of treatment strategies for this tumor: specialized, multidisciplinary management and treatment that combines multiagent chemotherapy, surgery, and, if necessary, radiotherapy.^11^ These studies have defined different risk groups, allowing treatment intensity to be adapted to the risk, thereby limiting complications and treatment costs. The SIOP studies have demonstrated the value of preoperative chemotherapy^3^ to limit the risk of tumor rupture during surgery, to increase the percentage of stage I tumors requiring less aggressive treatments, and to limit the risks and complications associated with surgery.^8^

In most African countries, results of such treatment management are fragmentary^12-16^ or unknown. In 2001, the Groupe Franco-Africain d'Oncologie Pédiatrique (GFAOP) initiated a protocol that was based on the SIOP 2001 study to test its feasibility in the African setting. Results show that participation in the study came predominantly form North African pilot units.^12^

The second GFAOP study (NEPHRO-02) was also a nonrandomized single-arm study that used the same treatment protocol to evaluate the overall survival (OS) of patients with unilateral WT that was limited to low- and intermediate-risk groups and that included only patients with stage IV disease in remission after surgery. Another aim was to evaluate the progress in existing units and assess the capacity of new units to follow the protocol, which is not too expensive and currently needs little supportive. This study was conducted in both North African and sub-Saharan pediatric oncology units. Here, we report the results of the sub-Saharan units. Results for the North African units will be presented later.

## PATIENTS AND METHODS

The protocol was reviewed and approved by the GFAOP board. Pediatric oncology units that were authorized to participate in the GFAOP-NEPHRO-02 study had to have a multidisciplinary team that was comprised of at least one pediatric oncologist, a surgeon, a pathologist, and a nurse. The team was collectively committed to present the protocol to their institution, to adhering to the protocol, to recording all patients with renal tumors who were admitted to their units, filling in data registration forms, and sending written surgical and pathology reports as well as pathology slides to the data center in Paris, France. A clinical research assistant was allocated to the study in four units during the study. Parents were informed and gave their consent for children to participate in the study.

### Patients

All patients who were admitted to the units with a clinical and radiologic diagnosis of a renal tumor were screened. Criteria for noninclusion were age younger than 6 months and older than 18 years, bilateral tumor, previously treated disease, a general condition that contraindicated chemotherapy, or relapse. Subsequently, after surgery and in accordance with the protocol as the intensified chemotherapy was not possible in these units, patients with high-risk renal tumors (clear-cell renal sarcoma, rhabdoid tumors, and WT with diffuse anaplasia or predominantly blastemal component)^17^ were not included in the study, nor were patients whose initial metastases had not been eradicated by preoperative chemotherapy, associated or not with metastasis resection.^18^

All patients who were pre-included underwent a complete clinical examination with measurement of the abdominal mass and biologic tests, including a CBC and serum electrolytes/creatininemia/transaminases and serology testing (hepatitis and HIV). An abdominal ultrasound and chest X-ray (with antero-posterior/lateral views recommended) were systematically performed. Abdominal and/or thoracic computed tomography scans were recommended only in the case of a doubtful diagnosis or suspicion of pulmonary metastases.

### Treatment

Pre-included patients received the same treatment as in the previous GFAOP-NEPHRO-01 protocol^12^ that was based on the SIOP2001 protocol which consisted of primary chemotherapy adapted to the initial disease extent. Drugs were provided by GFAOP and families paid for all other medical care.

Patients with localized disease received 4 weeks of preoperative chemotherapy combining vincristine with actinomycin. Additional treatment could be administered if surgery was delayed for any reason. Patients with stage IV disease received 6-week preoperative chemotherapy combining vincristine and actinomycin with doxorubicin.

Upon completion of preoperative chemotherapy, patients underwent a checkup, including an abdominal ultrasound and chest X-ray if initial pulmonary metastases.

The study recommended performing nephrectomy 1 week after the last course of chemotherapy using an abdominal transperitoneal approach, with exploration of the abdominal cavity, liver, and regional and distant lymph nodes.

The pathologist examined the nephrectomy specimen to confirm diagnosis, specify the histologic type, and establish the definitive disease stage. In the case of disagreement between the pathologist and surgeon, the pathologic diagnosis took precedence over the surgical stage. Both staging and histologic classifications are SIOP classifications.^11,17^ With respect to histologic classification, only WT excluding blastemal type and diffuse anaplastic forms were considered in this study. The low-risk group was treated similarly to the intermediate-risk group and both were grouped as standard risk.

Postoperative treatment was based on the SIOP2001 protocol with a few adjustments for fear of underestimating disease stage as in the previous GFAOP-NEPHRO-01 protocol.^12^ Patients with stage I disease received vincristine and actinomycin over 9 weeks rather than 4 weeks. Patients with stage II, III, or IV disease received postoperative chemotherapy that combined vincristine, actinomycin, and doxorubicin for a total duration of 27 weeks. Radiotherapy that was recommended for stage III tumors was not available in sub-Saharan units.

### Data Collection and Analysis

Data for each patient were sent to the study manager at Gustave Roussy (Villejuif, France) using questionnaires that summarized the main clinical, radiologic, surgical, and pathologic data; the chemotherapy administered; and information on the evolution of the tumor and survival. The histologic material received was to be sent for central review to Casablanca with an additional revision in Paris, France.

Data were reviewed at the study data management unit, then recorded and analyzed using Epi-info software. We estimated survival using the Kaplan-Meier method. For study patients, the date of origin was the date of nephrectomy, as currently used when reporting on WT. For OS, death was considered. Living patients were censored at the time of last follow up. In a first analysis, events considered for event-free survival (EFS) were relapse or death. In a second analysis, abandonment of treatment (defined as failing to complete therapy in a curable disease)^19^ was also considered an event.

## RESULTS

Seven pilot units participated in the study. In the previous GFAOP-NEPHRO-01 study, Dakar actively participated; however, Antananarivo and Yaoundé included only two patients each. This GFAOP-NEPHRO-02 study was the first in which Abidjan, Bamako, Lomé, and Ouagadougou participated. For all participating hospital pharmacies, drug provision, especially of actinomycin, was erratic and occasionally unavailable, despite drug delivery by GFAOP.

### Patient Inclusion

**FIG 1:**
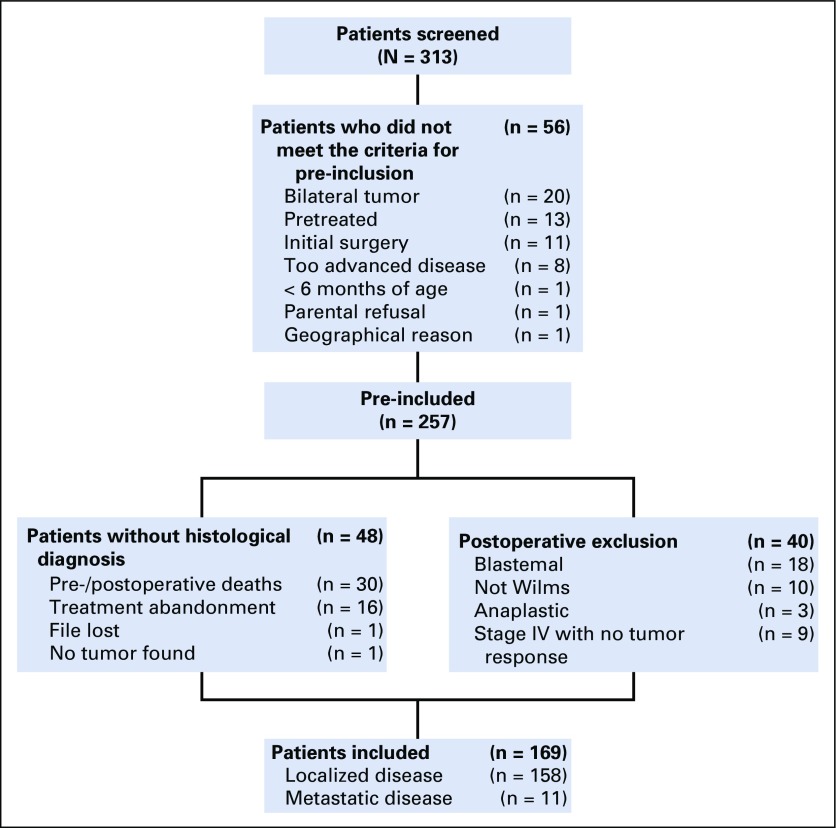
Flowchart of registered patients. The 30 pre-/peroperative deaths were a result of treatment-related toxicity (n = 8), tumor progression before any resection (n = 15), during surgery (n = 3), after surgery but without histologically examination (n = 3), and anesthetic accident (n = 1). Diagnosis of the 10 non-Wilms tumor cases excluded postoperatively were clear-cell sarcoma (n = 5), renal carcinoma (n = 2), Burkitt lymphoma (n = 1), neuroblastoma (n = 1), and bilharzia (n = 1). Slides reviewed in four cases confirmed the diagnoses of Burkitt (n = 1) and clear-cell sarcoma (n = 3). Among the 21 high-risk Wilms tumors, review confirmed the diagnosis of blastemal type in two cases and of diffuse anaplastic in two cases.

From April 1, 2005, to March 31, 2011, 313 patients were screened (Fig 1). Fifty-six patients were not pre-included because they did not fulfill protocol criteria. Among pre-included patients, 48 had no histologic diagnosis and 40 were excluded after surgery for histologic diagnosis other than standard-risk WT or for nonresponding metastasis as foreseen in the protocol. Thus, 169 children were retained for study analysis (clinical characteristics are listed in Table 1) and were administered postoperative treatment. One hundred fifty-eight children had localized tumors and 11 had metastatic tumors.

**TABLE 1:**
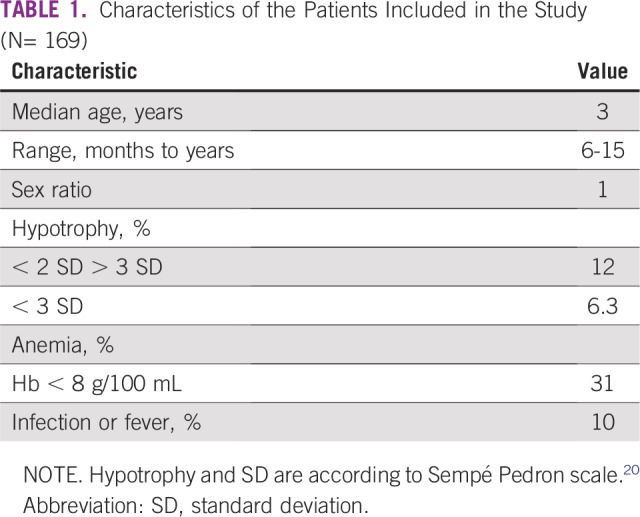
Characteristics of the Patients Included in the Study (N= 169)

### Quality of Data

Questionnaires on the initial clinical description and treatment were correctly completed and returned in all but two cases. Sixty-two percent of pathology and 90.5% of surgical questionnaires were returned for entry into the database. Furthermore, despite repeated requests, written reports were often missing or noncontributive, which did not permit validation of the staging. Pathology slides were sent for review for only 40 of 209 children who underwent surgery and concerned 32 of the 169 patients included in the analyses

### Protocol Compliance

#### Preoperative chemotherapy.

Preoperative chemotherapy was administered over 4 weeks in 120 patients (76%) and over 6 to 10 weeks in 17 of the 158 patients with localized disease. An additional 17 children received vincristine only. Information was incomplete for four children. Among the 11 patients with metastatic disease, eight received chemotherapy according to protocol and three received only vincristine and doxorubicin.

#### Nephrectomy, histology, and staging.

Nephrectomy was performed within an interval of fewer than 15 days after the end of preoperative chemotherapy (range, 1 to 28 days) in 89 (53%) of 169 patients. Local stage distribution is described in Table 2.

**TABLE 2:**
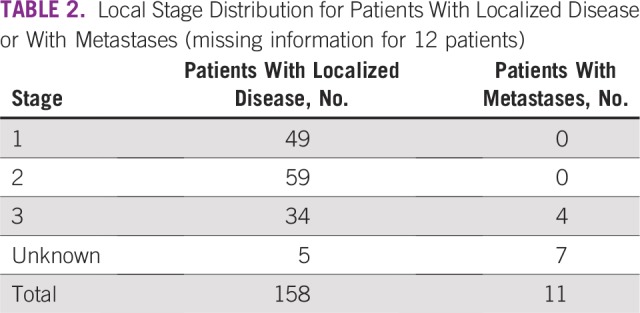
Local Stage Distribution for Patients With Localized Disease or With Metastases (missing information for 12 patients)

Among the 158 patients with localized disease, surgical and histologic reports were not available for 14 of 34 patients with stage III tumors. Tumor rupture was reported in 19 patients (12%); however, the rupture could not be confirmed in most cases because of the absence and or nonconcordance of written surgical and histologic reports.

#### Postoperative treatment.

No information on treatment was obtained for 18 patients with localized disease, although follow up was known for nine. Postoperative chemotherapy was administered according to the protocol for 76 (73%) of 104 patients who received the whole or major part of treatment. Among the 33 patients with stage I disease, 18 received vincristine and actinomycin according to the protocol, two received vincristine and doxorubicin (actinomycin was not available), and 15 patients received the three-drug schedule as a result of fear of understaging. Among the 64 patients with stage II and III disease, 58 received protocol chemotherapy, five received vincristine and doxorubicin, and one vincristine only. Compliance was not evaluable in four patients with unknown stage. For 13 children who received the correct drug combination, the number of courses administered was not documented. Among the 11 patients with metastasis, two had missing data on postoperative treatment, eight received the three drugs, and one received actinomycin and doxorubicin.

### Follow-Up

**FIG 2:**
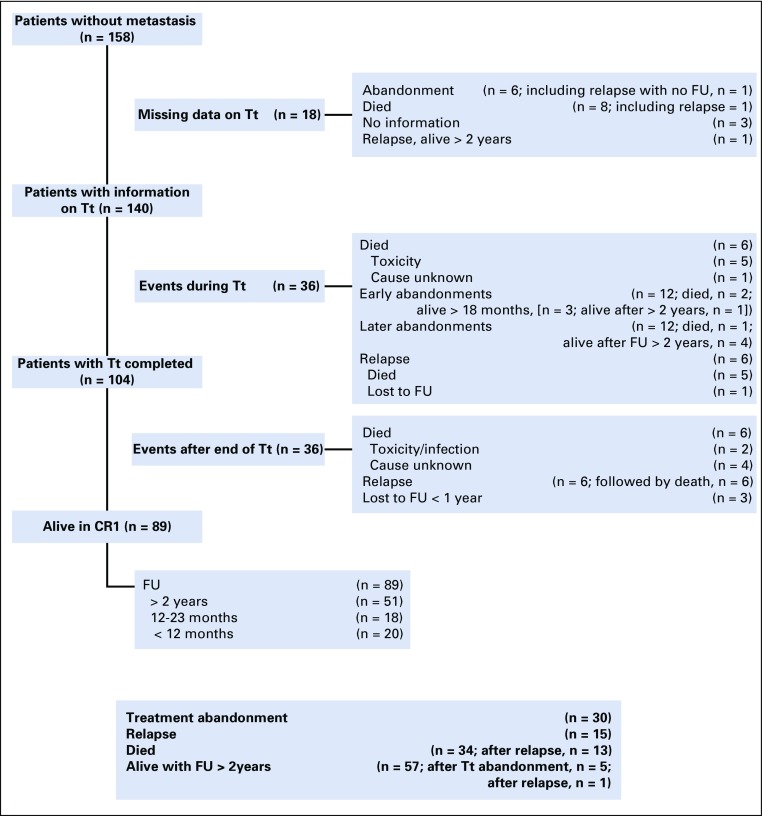
Outcome of the 158 patients with a nonmetastatic unilateral standard-risk histology Wilms tumor included in the study. Early abandonment is defined as during the 8-week postoperative phase; late abandonment defined as during maintenance treatment. CR1, in first complete remission; FU, follow up; Tt, treatment.

Median follow up of patients with localized disease was 2.7 years (Fig 2). Eighty-nine patients were in complete remission at the end of treatment. Thirty-four children died, seven of treatment-related toxicity, 13 after relapse, and 14 of undocumented deaths at home.

A total of 15 patients experienced relapse. The most frequent relapse site was the abdomen (59% of cases), followed by the lungs (41%), as shown in Table 3. Two children are long-term survivors.

**TABLE 3:**
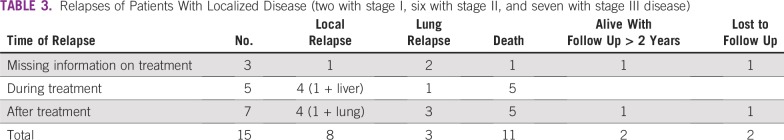
Relapses of Patients With Localized Disease (two with stage I, six with stage II, and seven with stage III disease)

Thirty patients (19%) abandoned treatment—one experienced relapse but had no follow up, three died, and seven are living with a follow up of more than 18 months. A total of 57 patients had a follow up superior to 2 years—51 in first remission after the completion of treatment, five after treatment abandonment, and one after relapse. Among the 34 patients who were considered locally as having stage III disease, eight are living with a follow up of more than 2 years, including two with tumor rupture, but four patients had no histologic report.

Among the 11 children with metastatic disease, there is no information for two. For the remaining nine children, one died during postoperative treatment, one experienced relapse, five abandoned treatment, and two were alive with 12 and 24 months follow up.

### Survival

**FIG 3:**
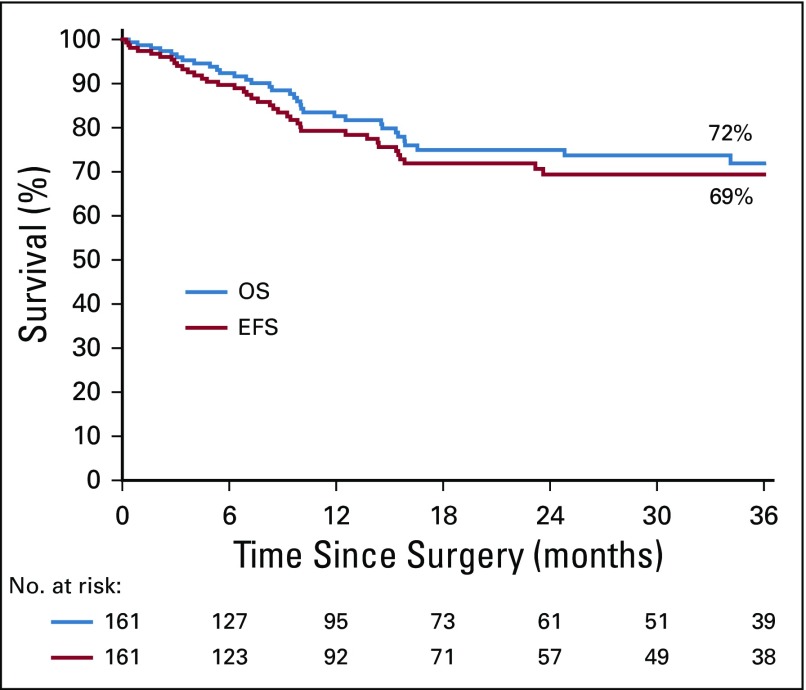
Overall survival (OS) and event-free survival (EFS) curves of the entire study population.

**FIG 4:**
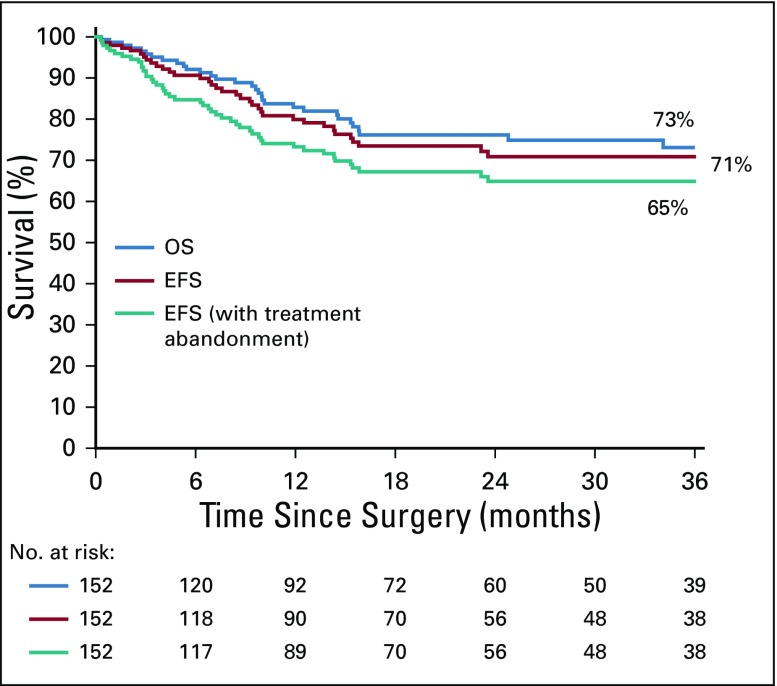
Survival curves of patients with nonmetastatic disease. OS, overall survival; EFS, event-free survival.

The date of surgery or the date of follow up is missing for eight children, giving a total of 161 children (153 without metastases and eight with metastases) who figure on the survival curves. OS at 3 years was 72% and EFS was 69% for all 161 children (Fig 3). For patients with localized disease, 3-year OS was 73%, and EFS was 71% and 65% when treatment abandonment was included in the list of events (Fig 4). The EFS rate was 75% for patients with stage I and II disease, and 43% for those with stage III disease.

## DISCUSSION

The first GFAOP-NEPHRO study, predominantly conducted in North Africa, demonstrated that a multicenter prospective study was feasible in Africa.^12^ This second GFAOP-NEPHRO study, to our knowledge, is the first multicenter prospective clinical WT study in sub-Saharan Africa with detailed analyses of treatment, staging, and outcome for such a large population. Despite many difficulties, it demonstrated that a multicenter study was possible within sub-Saharan countries, making possible a survival rate of 73% for patients with a documented unilateral localized standard-risk WT. Of the seven pilot units, it is important to note that for four units this was the first attempt at participating in a clinical oncology trial with the obligations of a multicenter international study, strictly following a protocol with multidisciplinary involvement and with prospective registration of detailed information for data processing for statistical analysis. Units were heterogeneous with regard to the number of patients registered and availability of chemotherapy, particularly actinomycin, and the programming of surgery and pathologic review were problematic for all participants.

Data collection was insufficient with respect to the written surgical and pathology reports as well as for postoperative chemotherapy forms. To improve the organization of pathologic review and the dispatch of pathology documents identified in the first study, three educative review panel meetings were organized and reviewing pathologists regularly attended SIOP review meetings in Paris, France. However, despite these efforts, the study remained lacking with regard to the quantity and quality of documented local initial staging and the transmitted documents for review. A reason for these shortcomings included the lack of a multidisciplinary approach. Units were not accustomed to submitting themselves to the demands of prospective, detailed, and critical data collection in a standardized manner; the workload entailed; and the distribution of tasks between the various players. Units that had a clinical research assistant allocated to the study were more efficient. For two units, this efficiency can also be attributed to previous participation in at least one GFAOP study.

Among the patients who were initially registered by participating units, 15% (11% in the first study) did not receive optimal therapy. This was a result of either death before surgery (linked to a precarious general health status and advanced disease, sometimes associated with treatment toxicity) or patients abandoned the treatment before surgery. Wilde^21^ relates the same difficulties in which more than one half of patients were malnourished with anemia. The 48 patients who were pre-included in the study and who withdrew before surgery demonstrate the real difficulties encountered in treating children with cancer in countries where the level of education is low and morbidity as a result of poverty is high. These findings are confirmed by the studies of Wilde in Malawi^21^, Yifru in Ethiopia^22^, Njuguna,^23^ and Kanyamuhunga in Rwanda^24^, but not in South Africa.^25^ Because units were obliged to declare all patients who presented with a renal tumor at the time of first consultation, patients who died or abandoned treatment before nephrectomy were included in our screened group of 313 patients. Such patients do not appear in studies in which registration is done after nephrectomy.

Whereas preoperative treatment was correctly administered in most cases, the main difficulties encountered in the application of the postoperative protocol concerned the availability of pathology reports within the protocol time range, availability of actinomycin, and the lack of access to radiotherapy. The interval between the end of preoperative chemotherapy and surgery and between surgery and the start of postoperative therapy was not always respected, often because the surgeon was not available or as a result of organizational difficulties and delayed pathologic reports. The impact of each of these problems on survival proved complicated to analyze, especially as considerable overlapping and clean data were not always available. Furthermore, this demonstrates again the need for a multidisciplinary approach as identified in the first GFAOP WT study.^12^ This situation has been described in other African countries, which, to varying degrees, have similar socioeconomic problems.^15,16^

The importance of the role of radiotherapy in local control and survival was reported for patients with stage III disease.^26-29^ In our series, study patients with a stage III tumor were denied proper protocol treatment because of a lack of access to radiotherapy. Initially, there was hope that children who required radiotherapy could be transferred to countries in which radiotherapy was available, but this proved difficult to do during the study. The authors are cautious about the interpretation of the outcome of the living patients with stage III disease as written surgical and pathologic reports were not submitted for review for all of these patients. There is little documentation on tumor rupture in the sub-Saharan series,^21,23,25,30,31^ likely because surgeons are seldom included in multidisciplinary discussions and are thus unaware of the consequences of rupture.

Postoperative treatment compliance was superior to that observed in other sub-Saharan studies,^21,23,24,30^ which could be attributed to organization and means implemented by the GFAOP. This could also be the result of the known phenomenon of amelioration observed related to the mere fact of participation in a study. It is the same observation as that which Israels et al^32^ reported for children included in a prospective multicenter clinical study that described the status at the end of treatment with a decrease in treatment abandonment.

Our OS of 73% is encouraging and, to our knowledge, is the best observed in any multicenter setting in sub-Saharan Africa.^22,24,30^ Nevertheless, caution must be taken when interpreting these results because of the high number of preoperative deaths.

One of the objectives of this study was to evaluate improvement in respecting procedures, data collection, and outcome; however, limited participation of two sub-Saharan units in the initial study, coupled with the addition of new participating units, makes this objective difficult to evaluate. The opening of a third WT study using an electronic case report form in units with the experience of at least one GFAOP study will allow for the evaluation of this objective in the future.

There is concern about the number of preoperative deaths in our first study and again in this study, which has been a motivating factor for a third study with adjustments for children with a precarious general health status. The adaptation of this new protocol for the treatment of patients with stage III disease, especially concerning radiotherapy in the sub-Saharan GFAOP units, remains a challenge for the future.

Striving for better multidisciplinary team organization, better adapted care, and better outcomes in the participating units is why GFAOP clinical research studies must continue in sub-Saharan Africa. Furthermore, given these encouraging results, the future study to open in early 2019 will exclude doxorubicin for patients with stage II disease and stage III disease for whom radiotherapy will be made available.^33^
